# Supplementary material for: Anthelmintic efficacy of Holarrhena pubescens against Raillietina spp. of domestic fowl through ultrastructural, histochemical, biochemical and GLCM analysis
Source: PLoS One. 2023 Sep 14;18(9):e0282033. doi: 10.1371/journal.pone.0282033 (PMC10501554; doi:10.1371/journal.pone.0282033)

PLOS ONE

Anthelmintic efficacy of <i> Holarrhena pubescens </i> against <i> Raillietina </i> spp. in domestic fowl through ultrastructural, histochemical, biochemical and GLCM analysis

--Manuscript Draft--

**Manuscript Number:**	PONE-D-23-03097
**Article Type:**	Research Article
**Full Title:**	Anthelmintic efficacy of <i> Holarrhena pubescens </i> against <i> Raillietina </i> spp. in domestic fowl through ultrastructural, histochemical, biochemical and GLCM analysis
**Short Title:**	Anthelmintic efficacy of <i> Holarrhena pubescens </i>
**Corresponding Author:**	Pradip Kumar Kar, Ph.D.<br>Cooch Behar Panchanan Barma University<br>Cooch Behar, West Bengal INDIA
**Keywords:**	Anthelmintic, SEM, TEM, Tegumental Enzyme, GLCM analysis
**Abstract:**	Holarrhenapubescens is a well-known medicinal plant used in ethno-traditional medicine and tribal folklore medicine in West Bengal, India. This article focuses on depicting the anthelmintic efficacy of ethanolic extract and Ethyl acetate fraction of the stem bark of Holarrhenapubescens against the cestode Raillietina spp. through efficacy testing, ultra-structural observations, histochemical and biochemical studies on some tegumental enzymes i.e., Acid Phosphatase (AcPase), Alkaline Phosphatase (AlkPase), Adenosine Triphosphatase (ATPase) and 5'-Nucleotidase (5'-Nu) along with Gray Level Co-occurrence Matrix (GLCM) analysis of histochemistry images. Praziquantel was used as the reference drug. Investigations revealed 10mg/ml of crude extract was determined as the most efficacious dose and the Ethyl acetate fraction showed the most anthelmintic property. Ultrastructural studies through Scanning Electron Microscope (SEM) and Transmission Electron Microscope (TEM) clearly showed the damage in head, sucker, proglottids, proximal and distal cytoplasm (DC), microtriches (MT), basal lamina (BL), nuclear membrane (NM), and, nucleolus (NL) in the treated worms. Histochemical studies revealed decrease in staining intensity for all the tegumental enzymes in the treated worms compared to control. The GLCM analysis strongly supported the result of histochemical studies. Biochemical studies revealed marked reduction in enzyme activity in the treated worms with maximum reduction in the activity of 5'- Nu (77.8%) followed by ATPase (63.17%).
**Order of Authors:**	Rachita Saha, M.Sc
	Manjil Gupta, M.Sc
	Rima Majumdar, M.Sc
	Pradip Kumar Kar, Ph.D
**Additional Information:**	
**Question**	**Response**
**Financial Disclosure**<br><br>Enter a financial disclosure statement that describes the sources of funding for the work included in this submission. Review the submission guidelines for detailed requirements. View published research articles from *PLOS ONE* for specific examples.<br><br>This statement is required for submission and **will appear in the published article** if	The authors received no specific funding for this work.

Powered by Editorial Manager® and ProduXion Manager® from Aries Systems Corporation

the submission is accepted. Please make sure it is accurate.<br><br>**Unfunded studies**<br>Enter: *The author(s) received no specific funding for this work.*<br><br>**Funded studies**<br>Enter a statement with the following details:<br>• Initials of the authors who received each award<br>• Grant numbers awarded to each author<br>• The full name of each funder<br>• URL of each funder website<br>• Did the sponsors or funders play any role in the study design, data collection and analysis, decision to publish, or preparation of the manuscript?<br>• **NO** - Include this sentence at the end of your statement: *The funders had no role in study design, data collection and analysis, decision to publish, or preparation of the manuscript.*<br>• **YES** - Specify the role(s) played.<br><br>* typeset	
**Competing Interests**<br><br>Use the instructions below to enter a competing interest statement for this submission. On behalf of all authors, disclose any competing interests that could be perceived to bias this work—acknowledging all financial support and any other relevant financial or non-financial competing interests.<br><br>This statement is required for submission and **will appear in the published article** if the submission is accepted. Please make sure it is accurate and that any funding sources listed in your Funding Information later in the submission form are also declared in your Financial Disclosure statement.<br><br>View published research articles from *PLOS ONE* for specific examples.	The authors have declared that no competing interests exist.

**NO authors have competing interests**<br><br>Enter: *The authors have declared that no competing interests exist.*<br><br>**Authors with competing interests**<br><br>Enter competing interest details beginning with this statement:<br><br>*I have read the journal's policy and the authors of this manuscript have the following competing interests: [insert competing interests here]*<br><br>* typeset	
**Ethics Statement**<br><br>Enter an ethics statement for this submission. This statement is required if the study involved:<br><br>• Human participants<br>• Human specimens or tissue<br>• Vertebrate animals or cephalopods<br>• Vertebrate embryos or tissues<br>• Field research<br><br>Write "N/A" if the submission does not require an ethics statement.<br><br>General guidance is provided below. Consult the submission guidelines for detailed instructions. **Make sure that all information entered here is included in the Methods section of the manuscript.**	The research involves collection and treatment of parasites from the intestine of fowls slaughtered for meat in the local market. So, approval from IACUC or other relevant ethics board is not required to the best of our knowledge.

Powered by Editorial Manager® and ProduXion Manager® from Aries Systems Corporation

**Format for specific study types**<br><br>**Human Subject Research (involving human participants and/or tissue)**<br>• Give the name of the institutional review board or ethics committee that approved the study<br>• Include the approval number and/or a statement indicating approval of this research<br>• Indicate the form of consent obtained (written/oral) or the reason that consent was not obtained (e.g. the data were analyzed anonymously)<br><br>**Animal Research (involving vertebrate animals, embryos or tissues)**<br>• Provide the name of the Institutional Animal Care and Use Committee (IACUC) or other relevant ethics board that reviewed the study protocol, and indicate whether they approved this research or granted a formal waiver of ethical approval<br>• Include an approval number if one was obtained<br>• If the study involved *non-human primates*, add *additional details* about animal welfare and steps taken to ameliorate suffering<br>• If anesthesia, euthanasia, or any kind of animal sacrifice is part of the study, include briefly which substances and/or methods were applied<br><br>**Field Research**<br><br>Include the following details if this study involves the collection of plant, animal, or other materials from a natural setting:<br>• Field permit number<br>• Name of the institution or relevant body that granted permission	
**Data Availability**<br><br>Authors are required to make all data underlying the findings described fully available, without restriction, and from the time of publication. PLOS allows rare exceptions to address legal and ethical concerns. See the PLOS Data Policy and FAQ for detailed information.	Yes - all data are fully available without restriction

A Data Availability Statement describing where the data can be found is required at submission. Your answers to this question constitute the Data Availability Statement and **will be published in the article**, if accepted.<br><br>**Important:** Stating 'data available on request from the author' is not sufficient. If your data are only available upon request, select 'No' for the first question and explain your exceptional situation in the text box.<br><br>Do the authors confirm that all data underlying the findings described in their manuscript are fully available without restriction?	
**Describe where the data may be found in full sentences. If you are copying our sample text, replace any instances of XXX with the appropriate details.**<br><br>• If the data are **held or will be held in a public repository**, include URLs, accession numbers or DOIs. If this information will only be available after acceptance, indicate this by ticking the box below. For example: *All XXX files are available from the XXX database (accession number(s) XXX, XXX.).*<br>• If the data are all contained **within the manuscript and/or Supporting Information files**, enter the following: *All relevant data are within the manuscript and its Supporting Information files.*<br>• If neither of these applies but you are able to provide **details of access elsewhere**, with or without limitations, please do so. For example:<br><br>*Data cannot be shared publicly because of [XXX]. Data are available from the XXX Institutional Data Access / Ethics Committee (contact via XXX) for researchers who meet the criteria for access to confidential data.*<br><br>*The data underlying the results presented in the study are available from (include the name of the third party*	All relevant data are within the manuscript.

and contact information or URL).<br>• This text is appropriate if the data are owned by a third party and authors do not have permission to share the data.<br><br>* typeset	
Additional data availability information:	Tick here if your circumstances are not covered by the questions above and you need the journal’s help to make your data available.

Powered by Editorial Manager® and ProduXion Manager® from Aries Systems Corporation

Manuscript
Click here to access/download;Manuscript;Saha et al. (Edited) manuscript.doc

Anthelmintic efficacy of *Holarrhena pubescens* against *Raillietina* spp. in domestic fowl through ultrastructural, histochemical, biochemical and GLCM analysis

RachitaSaha[1], Manjil Gupta[1], Rima Majumdar[1], Pradip Kumar Kar[1]*

[1] Parasitology Laboratory, Department of Zoology, Cooch Behar Panchanan Barma University, Cooch Behar, West Bengal, India
*karpradip@gmail.com

ORCID ids-
Rachita Saha- 0000-0001-6294-3555
Manjil Gupta- 0000-0001-9763-0217
Rima Majumdar- 0000-0001-8346-2072
Pradip Kumar Kar- 0000-0003-2656-6873

Abstract: *Holarrhena pubescens* is a well-known medicinal plant used in ethno-traditional medicine and tribal folklore medicine in West Bengal, India**.** This article focuses on depicting the anthelmintic efficacy of ethanolic extract and Ethyl acetate fraction of the stem bark of *Holarrhena pubescens* against the cestode *Raillietina* spp. through efficacy testing, ultra-structural observations, histochemical and biochemical studies on some tegumental enzymes i.e., Acid Phosphatase (AcPase), Alkaline Phosphatase (AlkPase), Adenosine Triphosphatase (ATPase) and 5ˈ-Nucleotidase (5ˈ-Nu) along with Gray Level Co-occurrence Matrix (GLCM) analysis of histochemistry images. Praziquantel was used as the reference drug. Investigations revealed 10mg/ml of crude extract was determined as the most efficacious dose and the Ethyl acetate fraction showed the most anthelmintic property. Ultrastructural studies through Scanning Electron Microscope (SEM) and Transmission Electron Microscope (TEM) clearly showed the damage in head, sucker, proglottids, proximal and distal cytoplasm (DC), microtriches (MT), basal lamina (BL), nuclear membrane (NM), and, nucleolus (NL) in the treated worms. Histochemical studies revealed decrease in staining intensity for all the tegumental enzymes in the treated worms compared to control. The GLCM analysis strongly supported the result of histochemical studies. Biochemical studies revealed marked reduction in enzyme activity in the treated worms with maximum reduction in the activity of 5ˈ- Nu (77.8%) followed by ATPase (63.17%).

Keywords: Anthelmintic, SEM, TEM, Tegumental Enzyme, GLCM analysis

Declarations: The authors declare that this article is an original report of research work, which has not been submitted in any journal before. The experimental work is almost entirely our own work and the contributions have been mentioned clearly in acknowledgment.

Conflict of interest: The authors declare no competing interests.

Introduction:

Neglected Tropical diseases (NTDs) appear globally in about 149 different nations (Gupta et al. 2022), particularly in the less developed ones where socioeconomic status are severely compromised. NTDs - like helminthiasis - cause a greater global burden of ailments rather than more well-known illnesses like malaria and TB, because they are the most prevalent infectious agents affecting humans and animals in underdeveloped nations (Hotez et al. 2008). Currently, it is estimated that 33% of people living in the developing countries in Sub-Saharan Africa, Asia, and Americas are infected with helminths (Hotez and Herricks 2015). A previous study revealed that the average weight of flocks of domestic fowls with helminth infections was considerably lower than flocks without such infestations. Domestic poultry is the primary source of dietary protein in the world, providing both meat and eggs (Permin 2020). Particularly in underdeveloped nations, livestock producers sometimes lack to access basic veterinary service and medications, where medicinal plants continue to be utilised in concoction with veterinary pharmaceuticals to cure animal maladies (Tchetan et al. 2022).

Plant materials have been used by Indians as curatives for various infections/diseases of veterinary significance since times immemorial. *Holarrhena pubescens* is one of them which is used by local Rajbangshi or Koch community in Cooch Behar, West Bengal, India. *Holarrhena pubescens* (Buch. Ham)Wall. ex G. Don, Syn - *Holarrhena*. *antidysenterica* (Roth) Wall. ex A. DC. belongs to family Apocynaceae, (Common Name: *Indrajao, Kutraj, Kutaja*, Bitter Oleander, *Kurchi*) and is indigenous to the eastern part of Tropical Africa and Tropical Asia (Indo-China). It is distributed in the subtropical forests of India, Myanmar, Nepal, Bhutan, China and Malaysia (Zahara et al. 2020). *H. pubescens* is a large tree, 9-18 meter long usually found in plains and mountainous areas (Bhusal 2014). Various parts of the plant (stem, root, leaves, and flower) have been reported to have astringent, antimalarial, antidysenteric, anthelmintic, stomachic, febrifugal, antibacterial, and antioxidant properties. The methanolic extract of stem bark of this plant reported hypoglycaemic activity (Bhusal 2014).The current investigation focuses on the plausible anthelmintic property of the ethanolic extract of stem bark of *H. pubescens* through ultrastructural, histochemical, GLCM analysis and biochemical studies of the alterations in the activity of the tegumental enzymes of *Raillietina* spp. collected from *Gallus gallus domesticus,* available commercially.

Materials and Methods:

Preparation of extract from *Holarrhena pubescens:*

Fresh bark (without chlorophyll) was collected from the plant *H. pubescens* from the town of Cooch Behar, West Bengal, India. The plant materials were finely chopped and air dried for 24-36 hours after being collected. The dried bark components were processed for preparation of ethanolic extracts for 15-20 days. The solution was then filtered using Whatman Filter Paper (No. 14), and then dried using a Rotary Vacuum Evaporator (Buchi Rotavapor R-100). After drying, the collected crude extracts of plant material were stored in a container at 4°C until further use. Around 7.0-8.0 gram (g) of plant extract was obtained from 100 g of plant material immersed in 500 millilitre (ml) ethanol. Using a separating funnel and fractional distillation process, different fractions of the ethanolic extract were separated using solvents with varying polarities such as hexane, chloroform, ethyl acetate, and n-butanol (MK et al. 2012).

Collection of Parasites:

Fresh intestines of *Gallus gallus domesticus* were obtained from a local market, and live parasites (*Raillietina* spp.) were isolated from the gut in 0.9 percent Phosphate-Buffered Saline (PBS: NaCl-8g, KH_2PO_4-0.34g, and $KHPO_4$-1.21g in 1 litre (l) distilled water, pH 7.4) and kept in an incubator set at 37 ± 1^0 C.

Experiments:

Efficacy Testing:

The model parasite i.e., *Raillietina* spp. were incubated at 37°C in PBS without plant extract (control), and with Ethanolic crude extract at 1.0, 2.0, 5.0, 10.0, 15.0 and 20.0 mg/ml concentration respectively. Praziquantel was used as the reference drug. Three replicates were used for each concentration. The time required for complete inactiveness or paralysis and death of the parasites were recorded. After determining the most efficacious dose of the crude extract, efficacy tests were performed with the same dose of different fractions.

Scanning Electron Microscopy:

For Scanning Electron Microscopy (SEM) the parasites were fixed in10% Neutral Buffered Formalin (NBF) at 4°C for 4 hours followed by repeated washing in double-distilled water, dehydration through acetone grades, critical-point-drying (CPD) using liquid CO_2, metal-coating with gold palladium, and viewed in a JEOL-JSM-35 CF scanning electron microscope (Roy and Giri 2015a).

Transmission Electron Microscopy:

Control and treated worms were fixed in Karnovosky's fixative (4% Paraformaldehyde and 1% Glutaraldehyde) in 0.1M Sodium Phosphate buffer (pH 7.4) and then processed for Transmission Electron Microscope TECNAI G20 HR-TEM as per usual method (Chaffey 2001) for ultrastructural studies (Roy and Giri 2015a).

Histochemical studies:

Following Pearse 1968, histochemical localisation of Acid Phosphatase (AcPase), Alkaline Phosphatase (AlkPase), and Adenosine Triphosphatase (ATPase) in control and phytoproduct exposed worms was carried out (Giri and Roy, 2014). The Wachstein and Meisel 1957 approach, which uses adenosine monophosphate as a substrate, was used to examine 5ˈ- Nucleotidase (5ˈ-Nu). For 30 minutes, sections were incubated at 37◦C in a freshly produced medium containing 10 ml of 1.25 percent adenosine-5-phosphate, 5 ml of 0.2 M Tris buffer at pH 7.2, 30 ml of 0.2 percent $Pb(NO_3)_2$, and 5 ml of 0.1 M $MgSO_4$. All parasites were cut in 12µm-14µm thickness through Cryostat (Leica- CM 3050S) at -15°C -18°C (Dey and Roy 2018a).

GLCM analysis:

Gray Level Co-occurrence Matrix (GLCM) analysis for the quantification of the histochemical localization of the enzymes was performed following Dragić et al. 2019. Mean Gray Value (MGV) and Integrated Density (IG) were calculated for all the images. Five parameters namely Angular Second Moment (ASM), Contrast (CON), Co-relation (COR), Inverse Different Moment (INV) and Entropy (ENT) were calculated for GLCM analysis. Out of these, ASM, COR and INV measures the homogeneity of the image and should be more in deeply stained images whereas CON and ENT measures contrast and lack of spatial organization in the image and should be less in deeply stained images. Images obtained from histochemical study for different enzymes were cropped for region of interest (comprising of the tegument, sub tegument and parenchyma), converted to grayscale and subjected to GLCM analysis through the software Image J (Abràmoff et al. 2004). A set of 20 images each from different categories like control, Praziquantel treated, crude extract treated and Ethyl acetate fraction treated was used for GLCM analysis for each enzyme. Receiver Operating Characteristic (ROC) curve analysis was also performed to validate the results obtained from GLCM analysis.

Biochemical studies:

The method of Plummer,1988, was used to estimate the activities of AcPase and AlkPase (Roy and Giri 2015a). ATPase activity was determined by estimating the free phosphate released using the method of Kaplan, (1957),

and 5' -Nu was determined by the Bunitian method following Giri and Roy, 2014. Protein content was calculated using Lowry's method (Lowry et. al. 1951) for all the enzyme assays. The total enzyme activity was determined for AcPase and AlkPase as the amount in micromole (μM) of p-nitrophenol formed per minute per gram wet tissue. In case of ATPase and 5'- Nu, activity was expressed by liberation of μM inorganic phosphate group per mg protein. The activity of tegumental enzymes was expressed by total and specific enzyme activity.

Results:

Efficacy Testing:

The results indicate that the Ethanolic crude extract of *Holarrhena pubescens* and the fractions of ethanolic extract showed anthelmintic activity on cestode worm *Raillietina* spp. In the presence of varying concentrations of the ethanolic extract and different fractions, it was observed that the survival time of the treated worms was longer than that of those treated with Praziquantel (PZ) but lower than the Control (C) (Table 1, Figure 1).The concentration of 10 mg/ml was determined as the most efficacious dose and used for further studies. Out of the different fractions used, the Ethyl acetate fraction (EAF) showed most anthelmintic activity.

Table1 Results of Efficacy testing of the ethanolic crude extract of *Holarrhena pubescens* and fractions of the ethanolic extract on *Raillietina* spp.

Incubation Medium	Dose (mg/ml) PBS	Time of paralysis (hours)	Time of death (hours)
**Control**	-	-	71.0±0.2
**Praziquantel**	0.05	0.48±0.38	0.80±0.16*
**Ethanolic Crude extract**	1.0	9.81±0.47	12.84±0.48*
	2.0	5.09±0.15	8.52±0.16*
	5.0	2.11±0.23	6.89±0.36*
	10.0	1.02±0.13	4.44±0.21*
**n-Hexane fraction**	10.0	6.16±0.42	20.23±0.33*
**Chloroform fraction**	10.0	5.32 ± 0.14	19.44±0.12*
**Ethyl acetate fraction**	10.0	0.83 ± 0.32	3.72±0.51*
**n-Butanol fraction**	10.0	1.08 ± 0.22	4.66±0.41*

*Data represents mean value ±SD. n= 450. Values significant at P<0.05

Fig.1 Graph showing the paralysis time and death time of *Raillietina* spp treated with different doses and fractions of the ethanolic extract of *Holarrhena pubescens.*

SEM Observations:

In the control worms, the entire surface of the scolex and proglottids shows a smooth appearance due to being covered with dense microtriches (Fig 2. a, e, i). Suckers are smooth with distinct hooks. In the worms treated with the plant material, distortions are visible in the head region characterised by folding of the tegument, eversion of the suckers and loss of the hooks encircling them. In the proglottids, the tegument shows a folded and cracked appearance (Fig 2. c, d, g, h, k, l). Similar observations were also found in the Praziquantel-treated worms (Fig 2. b, f, j). The damage is more apparent in the worms treated with Praziquantel and Ethyl acetate fraction.

Fig. 2 Scanning electron micrographs of the head (a - d), neck (e - h) and proglottids (i - l) of control and treated *Raillietina* spp. Control (a ,e ,i), Praziquantel exposed (b ,f ,j), Ethanolic crude extract exposed (c ,g ,k), Ethyl acetate fraction exposed (d ,h ,l) (all scale bars =20µm).

TEM Observations:

The control worm's surface, proximal and distal cytoplasm (DC) with microtriches (MT), basal lamina (BL) (Fig.3 a) nucleus with double nuclear membrane (NM), nucleolus (NL), chromatin granules (Fig.4 a, e), and mitochondrial characteristics were all normal, according to transmission electron microscopic studies. Parasites treated with Praziquantel had severe damage in the microtrix layer and distal cytoplasm (Fig.3 b). Swollen nuclei with an anomalous singlet nuclear membrane (Fig.4 b) and deformed mitochondria lacking cristae were also observed (Fig.4 f). The distal cytoplasm of the worms exposed to the plant extract (crude) was completely eroded, and, the basal lamina of the microtriches was exposed to the outer surface (Fig.3 c). The nucleolus disintegrated and a swollen nucleus with an uneven nuclear membrane was observed (Fig.4 c). In the parasites treated with the plant extract, the majority of the mitochondria developed deformities (Fig.4 g). *Raillietina* spp., treated with the Ethyl acetate fraction, similarly exhibited fully damaged microtriches and distal cytoplasm (Fig.3 d), rupture of the nuclear membrane, condensation of chromatin granules (Fig.4 d) and disintegration and vacuolization of the mitochondrial membrane (Fig. 4 h).

Fig. 3: Transmission electron microscopy images of sections of Control (a), Praziquantel (b), Ethanolic crude extract (c), Ethyl acetate fraction treated (d) *Raillietina* spp. showing Microtrix layer (MT), distal cytoplasm (DC) and basal lamina (BL) (all scale bar=1µm)

Fig. 4: Transmission electron microscopy images of sections of Control (a, e), Praziquantel treated (b, f), Ethanolic crude extract (c, g) Ethyl acetate fraction (d, h) treated Raillietina spp showing nucleus (N), nucleolus (NL) and nuclear membrane (NM) and mitochondria (M); Scale bars=0.5 µm (a - d) and 0.2 µm (e - h).

Histochemical Observation:

The tegument of control *Raillietina* spp. showed intense activity of AcPase, AlkPase, ATPase and 5' Nu compared to sub-tegument, somatic musculature, and parenchyma (Figs. 5 a - d; e - h; 6 a - d; e - h). ATPase activity was almost negligible in the tegument and sub-tegument of the parasite treated with crude and Ethyl acetate fraction of the plant extract compared to control (Fig. 6 c, d). A reduced activity of AlkPase was observed throughout the treated sections of the parasite (Fig. 5 g, h). In the control sections of histochemical localization of AcPase, pronounced stain intensity was observed in the tegument and sub- tegument regions (Fig. 5 a - d). However, there was minimal AcPase activity oberved throughout the section of parasite exposed to crude, Ethyl acetate fraction and Praziquantel. The 5' -Nu activity was also found to be reduced throughout the tegumental and sub-tegumental region in the phytoproducts exposed worms compared to the control (Fig. 6. e - h).

Fig. 5: Histochemical evidence of AcPase (a - d) and AlkPase (e - h) activity in *Raillietina* spp., T-Tegument, ST -Subtegument, SM-Somatic musculature, P-Parenchyma, (a , e). Control, (b , f) Praziquantel, (c , g) Crude extract (d , h) Ethyl acetate fraction treated (All scale bars =50 µm)

Fig. 6: Histochemical evidence of ATPase (a - d) and 5'-Nu (e - h) activity in *Raillietina* spp., T-Tegument, ST - Subtegument, SM-Somatic musculature, P-Parenchyma, (a , e) Control, (b , f) Praziquantel, (c , g) Crude extract treated, (d & h) Ethyl acetate fraction treated (All scale bars =50 µm)

GLCM results:

The calculated values of different GLCM parameters are depicted in Table 2. For ASM, COR and INV, the values are more in the control worms than in the treated worms and for MGV, ID, CON and ENT the control worms show lower values than the treated worms. The results conform to the expected results. MGV and ID showed the lowest value in the control worms of AcPase due to darker staining section compared to the other enzymes. ASM and CON showed highest and lowest values respectively in the control worms for ATPase. COR shows highest vale in the control worms of AcPase. INV has highest value in the control worms of ATPase. ENT has lowest value in the control worms of ATPase. The ROC curve analysis is shown in Figure 7. The area

under the curve (AUC) values is depicted in Table 3. All the values are greater than 50% indicating that the process is considerable.

Table 2. GLCM parameters of images obtained from histochemical studies of different enzymes in *Raillietina* spp. C- Control, PZ-Praziquantel treated, ECE-Ethanolic Crude Extract treated, EAF- Ethyl acetate fraction treated.

		MGV	ID	ASM	CON	COR	INV	ENT
AcPase	C	113.03 ±15.07	7064539.0 ±941926.3	0.000473 ±0.000112	189.95 ±58.36	0.0022 ±0.0006	0.13 ±0.0171	7.99 ±0.26
	PZ	*168.67 ±16.03	*10542149.4 ±1002093.42	*0.000172 ±0.000027	*558.7 ±66.56	*0.00081±0.00009	*0.07 ±0.0054	*8.95 ±0.12
	ECE	*136.82 ±31.29	*8551189.8 ±1955700.6	*0.000372 ±0.00015	*331.26 ±218.26	0.00172 ±0.00071	*0.10 ±0.0285	8.28 ±0.5
	EAF	*146.41 ±34.33	*9306178.5 ±2190156.44	*0.000299 ±0.000153	*433.75 ±239.09	*0.00137±0.00063	*0.09 ±0.0296	*8.53 ±0.54
AlkPase	C	160.11 ±3.18	15287542.5 ±303448.53	0.000248 ±0.000011	311.32 ±38.36	0.001±0.0	0.09 ±0.0431	8.59 ±0.05
	PZ	*164.71 ±5.4	*15726452.2 ±515784.83	0.000234 ±0.00004	*383.33 ±33.5	0.001±0.0	*0.08 ±0.0045	8.67 ±0.14
	ECE	*169.76 ±5.79	*15930277.4 ±671090.57	0.000242 ±0.000027	*411.49 ±55.56	0.00099±0.00002	*0.08 ±0.0073	*8.67 ±0.1
	EAF	*167.65 ±8.07	*15825408.9 ±715618.67	0.000236 ±0.000045	*445.2 ±56.4	*0.00099±0.00002	*0.08±0.01125	*8.71 ±0.12
ATPase	C	175.75 ±12.06	67556571.1 ±4636486.82	0.00079 ±0.000282	88.9 ±14.54	0.00157 ±0.00053	0.23 ±0.0432	7.59 ±0.43
	PZ	182.64 ±16.28	70208272.0 ±6259795.72	*0.00053 ±0.000124	*99.49 ±11.8	0.00128 ±0.00048	*0.14 ±0.0089	*7.96 ±0.18
	ECE	184.07 ±9.89	70534709.2 ±3908405.44	*0.00035 ±0.000071	*124.57 ±15.59	*0.00107±0.00032	*0.19±0.0101	*8.33 ±0.15
	EAF	183.12 ±7.26	70442003.2 ±2923379.36	*0.00034 ±0.000066	*118.6 ±12.49	*0.001±0.00034	*0.15 ±0.008	*8.35 ±0.13
5'- Nu	C	169.78 ±8.13	69543524.8 ±3329793.72	0.00028 ±0.00003	153.14 ±26.47	0.00098 ±0.00007	0.14±0.0127	8.53 ±0.1
	PZ	*19.37 ±6.13	*77976486.4 ±2509017.94	*0.0002 ±0.000031	*173.83 ±29.83	*0.00054±0.00008	*0.12 ± 0.0099	8.62 ±0.13
	ECE	*178.98 ±7.15	*73310255.7 ±2928377.66	*0.00023 ±0.000022	*190.3 ±24.62	*0.00083±0.00006	*0.12 ±0.0142	*8.68 ±0.08
	EAF	*178.65 ±6.34	*73228978.2 ±2717574.88	*0.00025 ±0.00004	*176.1 ±27.34	*0.0009±0.00009	*0.12±0.0132	*8.8 ±0.14

Data represent Mean±SD. * Values significant at P<0.05

Table 3. Area under curve (AUC) values of GLCM parameters of images obtained from histochemical studies of different enzymes in *Raillietina* spp. Mean Gray Value –MGV, Integrated Density – IG, Angular Second Moment - ASM, Contrast- CON, Co-relation - COR, Inverse Different Moment - INV and Entropy – ENT.

Enzyme	Area under curve (AUC) values (%)						
	MGV	ID	ASM	CON	COR	INV	ENT
AcPase	81.00	72.00	81.00	86.00	82.00	88.00	80.00
AlkPase	80.00	74.00	85.00	94.00	62.00	83.00	74.00
ATPase	69.00	68.00	87.00	88.00	74.00	63.00	87.00
5'- Nu	84.00	84.00	65.00	78.00	88.00	88.00	84.00

Fig. 7: ROC curves for GLCM parameters of images obtained from histochemical studies on different enzymes in *Raillietina* spp.

Biochemical observation:

The quantitative analysis of the tegumental enzymes of *Raillietina* spp. showed significant impact on treatment with the plant materials and the reference drug Praziquantel. As shown in results (Table 4 and Figure 8) AcPase activities decreased by 50.33, 39.70 and 60.56 percent, AlkPase activities decreased by 54.05, 65.44 and 39.74 percent, ATPase activities decreased by 37.16, 41.38 and 63.17 percent, and 5´-Nu activity decreased by 71.63, 65.75, and 77.80 percent, after exposure to the Praziquantel, Ethanolic crude extract, Ethyl acetate fraction of the crude extract respectively. In the worms fed with the Ethyl acetate fraction of the plant, the highest inhibition of activity was observed in the case of 5´- Nu followed by ATPase.

Table 4. Results of biochemical analysis of enzyme activity in *Raillietina* spp.

	Tegumental enzyme (Total/Specific) activity in µM				Percentage changes after treatment (%)			
	AcPase	AlkPase	ATPase	5´-Nu	AcPase	AlkPase	ATPase	5'- Nu
C	21.71± 1.99/ 9.07 ± 0.87	30.09±1.62/ 11.74±1.25	244.27±13.96/ 45.78±4.07	771.15±22.06/ 159.93±18.5	-	-	-	-
PZ	11.84±1.39/ *4.50±0.65	13.97±1.36/ *5.40±0.73	153.68±13.43/ *28.77±2.15	234.98±33.01/ *45.37±4.7	50.33	54.05	37.16	71.63
ECE	12.98±0.7/ *5.47± 0.76	21.35±1.74/ *8.21±0.99	150.25±11.9/ *26.84±2.83	287.6±30.35/ *54.78±7.85	39.7	65.44	41.38	65.75
EAF	8.33±1.09/ *3.58± 0.52	17.68±1.96 / *7.08±0.89	89.54±8.97/ *16.86±1.95	202.38±24.84/ *35.49±5.92	60.56	39.74	63.17	77.8

Data represents mean value ± SD. *Values are significant at $P \leq 0.05$.
C- Control, PZ- Praziquantel treated, ECE- Ethanolic crude extract treated, EAF- Ethyl acetate fraction treated.

Fig. 8: Graph showing the effect on total and specific activities of different tegumental enzymes of Raillietina spp. treated with crude extract and fractions of *Holarrhena pubescens.* C- Control, PZ- Praziquantel treated, ECE- Ethanolic crude extract treated, EAF- Ethyl acetate fraction treated.

Discussion

It is reported that the stem bark, root bark and seeds of *Holarrhena pubescens* has more than 40 alkaloids. The highest concentration of alkaloids, i.e., up to 4.3%, was found in the barks of stem and root (*in toto*) followed by the leaves 1.0–1.5% and seeds 0.6–1.8%. Of the 4.3% alkaloids found in the barks of stem and root, only 0.4% is available for flowering plants. According to the Ayurvedic Pharmacopoeia of India (Anonymous 2010), it is reported that the bark contained about 2% alkaloids. The stem bark, seeds, and roots of this plant were reported to be rich in steroidal alkaloid compounds such as asisoconessine, kurchine, conessidine, conkurchicine, and holarrhimine and conessine - which has antimalarial and antibiotic properties (Verma et al. 2011; Siriyong et al.

2018). Previous studies showed that the leaf, bark, fruit and seed of the plant has medicinal value. Leaf of the plant is used in pest management and the whole plant is used as green manure as well as in Ayurveda, Homoeopathy and Folklore medicine. The flavonoids and phenolic compounds of aqueous and methanolic bark extract of *Holarrhena pubescens* showed strong antioxidant property (Bhusal 2014). All the alkaloids of *H. pubescens* were found effective in treating acute dysentery, inflammatory bowel disease, diarrhoea and worm infections in the intestine (Bala et al. 2022). It is also reported that Kutaja, may be helpful to an extent, in treating Amoebiasis (Singh 1986) and this plant also has anti-diabetic, anti-amnesic and anti-inflammatory property (Bala et al. 2022).

In our present study, the ethanolic crude extract treated worms showed a dose dependent increase in the time of paralysis and death indicating the putative anthelmintic potential of *Holarrhena pubescens.* Amongst the fractions, the worms treated with the Ethyl acetate fraction showed the highest reduction in motility and survivability. The tegument in cestodes forms a digestive-absorptive-protective surface at the host parasite interface (Lumsden 1975, Thompson and Geary 1995). The action of most anthelmintic drugs depend on their passive transfer through this external surface i.e. the tegument (Alvarez et al. 2007). Ultrastructural studies (SEM) showed distortion of the smooth texture of the tegument as well as cracking and folding. This is likely to interfere with the nutrient uptake and energy metabolism of the parasite. Similar type of alterations/damages to the tegument by phytoproduct treated worms have been reported by many authors (Roy et al. 2007, 2008; Kar et al. 2014; Dey and Roy 2018b). The TEM images further show that the microtrich layer, nuclear membrane, chromatin as well as mitochondria, all suffer damages in the phytoproduct treated worms. This indicates the interference - of the phytoproducts - with the energy metabolism of the worms as well as functioning of the nucleus. In the present study, the tegumental enzymes namely Acid Phosphatase, Alkaline Phosphatase, Adenosine Triphosphatase and 5’-Nucleotidase show pronounced activity in the tegument, subtegument, somatic musculature and parenchyma of the control worms. However, in the phytoproduct as well as Praziquantel treated worms, there is a marked reduction in the activity of all the four enzymes in the aforementioned regions. Similar results were also evident from the biochemical studies wherein there is a marked reduction in the activity of the tegumental enzymes in the Praziquantel and phytoproduct treated worms. Acid Phosphatase is an important lysosomal enzyme in helminths (Smyth et al. 1989) and the reduction in its activity indicates the disruption of lysosomes in the phytoproduct treated worms (Tandon et al. 1997). Alkaline Phosphatase is essential for the uptake of glucose through the tegument (ROGERS 1947) and reduction in its

activity may interfere with the glucose uptake as well as energy metabolism in the worms (Roy and Giri 2015b; Dey and Roy 2018b). Adenosine Triphosphatase in helminths plays a role in the transport of various ions and phospholipids across the tegument (Burenina 2007) and reduction in its activity indicates that the crude plant extract as well as the Ethyl acetate fraction interferes with the transport of different ions as well as the energy metabolism of the worms. Reduction in 5'-Nu activity in the treated worms suggest interference with the uptake of nucleosides (Pal and Tandon 1998).

Texture analysis of images is used to quantify spatial differences in the pixels. Gray Level Co-occurrence Matrix (GLCM) is one such method of image texture analysis. It has been used for the enzyme histochemistry in neural tissues (Chen et al. 2021; Zaletel et al. 2021) and analysis of chromatin architecture (Paunovic et al. 2019). We, for the first time are reporting the use of GLCM for enzyme histochemistry analysis in invertebrates. The MGV represents the brightness of the image and the ID is equivalent to the product of the area and MGV. So, the values of these two parameters are lower in the control images as because the intensity of the staining is higher for all the enzymes in the control images compared to the treated images. ASM and INV measure tissue homogeneity and may increase due to the changes in tissue texture due to increased staining. In our analysis, both ASM and INV are higher in the control images with higher staining intensity than in the phytoproduct treated images. CON measures local differences in the image and ENT measures the lack of spatial organisation. Essentially, CON and ENT will increase with the decrease in image homogeneity. So, the values of CON and ENT are lower in the control images compared to the phytoproduct treated images of the worms. High COR values indicate that the pixels are dependent on one another and vice versa. We obtained slightly higher COR values in control images as compared to treated. From Table 2 it is evident that CON and INV are better indicators of the enzyme histochemistry results.

Conclusion

The results of efficacy and ultrastructural analysis clearly show that the stem bark Ethanolic crude extract and Ethyl acetate fraction of *Holarrhena pubescens* has anthelmintic potential. Histochemical and biochemical analysis has shown marked reduction in the activity of tegumental enzymes, i.e., AcPase, AlkPase, ATPase and 5´- Nu of the *Raillietina* spp. The result of various experiments has shown that the Ethyl acetate fraction of crude extract of stem bark of *H. pubescens* has higher effective anthelmintic property.

Acknowledgements

The authors gladly appreciate the constant support and help in conducting the experiments with instrumental and infrastructural facility of the Department of Zoology, Cooch Behar Panchanan Barma University, Cooch Behar. The authors are grateful to SAIF, North Eastern Hill University (NEHU), Shillong, for access to the SEM facility. We also acknowledge the TEM facility of SAIF, All India Institute of Medical Science (AIIMS), New Delhi for their kind cooperation.

Author Contribution

R.S. designed the study, performed all the experiments, collected and analysed the data and wrote the first draft of the manuscript.

M.G. carried out the GLCM analysis, interpreted the results and also helped in preparing the first draft of the manuscript.

R.M. helped in conducting the histochemical experiments.

P.K.K. designed the study and supervised all the experiments and finalised the draft of the manuscript.

Code availability: Not applicable.

Ethics approval: Not applicable.

Consent to participate: Not applicable.

Consent for publication: All the authors gave their consent for publication.

References:

Abràmoff MD, Magalhães PJ, Ram SJ (2004) Image processing with imageJ. Biophotonics Int 11:36–41. https://doi.org/10.1201/9781420005615.ax4

Alvarez LI, Mottier ML, Lanusse CE (2007) Drug transfer into target helminth parasites. Trends Parasitol 23:97–104. https://doi.org/https://doi.org/10.1016/j.pt.2007.01.003

Anonymous (2010) The Ayurvedic Pharmacopoeia of India, Part-I, V. Government of India, Ministry of Health and Family Welfare, Department of Ayurveda, Yoga-Naturopathy, Unani, Siddha & Homeopathy., New Delhi

Bala K, Melkani I, Singh AP, et al (2022) Holarrhena antidysenterica in Inflammatory Bowel Disease: a potential review. J Drug Deliv Ther 12:221–226. https://doi.org/10.22270/jddt.v12i4.5458

Bhusal A (2014) Evaluation of Antioxidative and Antidiabetic Activity of Bark of Holarrhena Pubescens Wall. J Clin DIAGNOSTIC Res. https://doi.org/10.7860/jcdr/2014/7803.4863

Burenina EA (2007) Adenosine triphosphatases of cestodes Bothriocephalus scorpii. J Evol Biochem Physiol 43:287–294

Chaffey N (2001) Hayat MA. 2000. Principles and techniques of electron microscopy: biological applications. 4th edn. 543pp. Cambridge: Cambridge University Press. £65 (hardback). Ann Bot 87:546–548. https://doi.org/10.1006/anbo.2001.1367

Chen H, Li W, Zhu Y (2021) Improved window adaptive gray level co-occurrence matrix for extraction and analysis of texture characteristics of pulmonary nodules. Comput Methods Programs Biomed 208:106263. https://doi.org/https://doi.org/10.1016/j.cmpb.2021.106263

Dey P, Roy B (2018a) Biochemical and ultrastructural changes in Raillietina echinobothrida in vitro exposed to extract of Lysimachia ramosa. J Parasit Dis 42:212–219. https://doi.org/10.1007/s12639-018-0985-z

Dey P, Roy B (2018b) Biochemical and ultrastructural changes in Raillietina echinobothrida in vitro exposed to extract of Lysimachia ramosa. J Parasit Dis 42:212–219. https://doi.org/10.1007/s12639-018-0985-z

Dragić M, Zarić M, Mitrović N, et al (2019) Application of Gray Level Co-Occurrence Matrix Analysis as a New Method for Enzyme Histochemistry Quantification. Microsc Microanal 25:690–698. https://doi.org/10.1017/S1431927618016306

Giri BR, Roy B (2014) Resveratrol induced structural and biochemical alterations in the tegument of Raillietina echinobothrida. Parasitol Int 63:432–437. https://doi.org/10.1016/j.parint.2013.12.008

Gupta S, Farina JM, Mendoza I, et al (2022) Chapter 2 - Malaria & Heart. In: Saldarriaga C, Baranchuk A (eds) Neglected Tropical Diseases and other Infectious Diseases affecting the Heart. Academic Press, pp 9–23

Hotez PJ, Brindley PJ, Bethony JM, et al (2008) Helminth infections: The great neglected tropical diseases. J Clin Invest 118:1311–1321. https://doi.org/10.1172/JCI34261

Hotez PJ, Herricks JR (2015) Helminth Elimination in the Pursuit of Sustainable Development Goals: A “Worm Index” for Human Development. PLoS Negl Trop Dis 9:1–6. https://doi.org/10.1371/journal.pntd.0003618

Kaplan C (1957) Methods in Enzymology. Academic Press, New York

Kar PK, Murmu S, Saha S, et al (2014) Anthelmintic efficacy of gold nanoparticles derived from a phytopathogenic fungus, Nigrospora oryzae. PLoS One 9:1–9. https://doi.org/10.1371/journal.pone.0084693

Lowry OH, Rosebrough NJ, Farr AL, Randall RJ (1951) Protein Measurement With The Folin Phenol Reagent. J Biol Chem 193:

Lumsden RD (1975) The Tapeworm Tegument: A Model System for Studies on Membrane Structure and Function in Host-Parasite Relationships. Trans Am Microsc Soc 94:501–507

MK S, WD N, SS O (2012) In-vivo evaluation for anthelmintic effect of alkaloids extracted from the stem bark of Afzelia africana in rats. J Adv Sci Res 100–104

Pal P, Tandon V (1998) Anthelmintic efficacy of Flemingia vestita (Leguminoceae): Genistein-induced alterations in the activity of tegumental enzymes in the cestode, Raillietina echinobothrida. Parasitol Int 47:233–243

Paunovic J, Vucevic D, Radosavljevic T, et al (2019) Gray-level co-occurrence matrix analysis of chromatin architecture in periportal and perivenous hepatocytes. Histochem Cell Biol 151:75–83

Pearse A (1968) Histochemistry: Theoretical and applied., 3rd edn. Churchill Livingstone, Edinburgh, London, New York.

Permin A (2020) Impact of Helminth Infections on Production of Chickens. Biomed J Sci Tech Res 29:22542–22550. https://doi.org/10.26717/bjstr.2020.29.004819

Plummer D (1988) An introduction to Practical Biochemistry. Tata-McGraw Hill, New Delhi.

ROGERS WP (1947) Histological Distribution of Alkaline Phosphatase in Helminth Parasites. Nature 159:374–375. https://doi.org/10.1038/159374b0

Roy B, Dasgupta S, Tandon V (2008) Ultrastructural observations on tegumental surface of *Raillietina echinobothrida* and its alterations caused by root-peel extract of *Millettia pachycarpa*. Microsc Res Tech 71:810–815. https://doi.org/10.1002/jemt.20623

Roy B, Giri BR (2015a) α-Viniferin-Induced Structural and Functional Alterations in Raillietina echinobothrida, a Poultry Tapeworm. Microsc Microanal 21:377–384. https://doi.org/10.1017/S1431927614014603

Roy B, Giri BR (2015b) α-Viniferin-Induced Structural and Functional Alterations in Raillietina echinobothrida, a Poultry Tapeworm. Microsc Microanal 21:377–384. https://doi.org/10.1017/S1431927614014603

Roy B, Lalchhandama K, Dutta BK (2007) Anticestodal efficacy of Acacia oxyphylla on Raillietin echinobothrida: A light and electron microscopic studis. Pharmacologyonline 287:279–287

Singh KP (1986) Clinical studies on amoebiasis and giardiasis evaluating the efficacy of kutaja (holarrhena antidysenterica) in entamoeba histolytica cyst passers. Anc Sci Life 5:228–31

Siriyong T, Voravuthikunchai SP, Coote PJ (2018) Steroidal alkaloids and conessine from the medicinal plant Holarrhena antidysenterica restore antibiotic efficacy in a Galleria mellonella model of multidrug-resistant Pseudomonas aeruginosa infection 11 Medical and Health Sciences 1108 Medical Microbiolo. BMC Complement Altern Med 18:1–10. https://doi.org/10.1186/s12906-018-2348-9

Smyth JD, McManus DP, others (1989) The physiology and biochemistry of cestodes. Cambridge university press

Tandon V, Pal P, Roy B, et al (1997) In vitro anthelmintic activity of root-tuber extract of Flemingia vestita, an indigenous plant in Shillong, India. Parasitol Res 83:492–8

Tchetan E, Olounladé PA, Azando EVB, et al (2022) Anthelmintic Activity, Cytotoxicity, and Phytochemical Screening of Plants Used to Treat Digestive Parasitosis of Small Ruminants in Benin (West Africa). Animals 12:. https://doi.org/10.3390/ani12192718

Thompson DP, Geary TG (1995) 12 - The Structure and Function of Helminth Surfaces. In: Marr JJ, Müller M (eds) Biochemistry and Molecular Biology of Parasites. Academic Press, San Diego, pp 203–232

Verma G, Dua VK, Agarwal DD, Atul PK (2011) Anti-malarial activity of Holarrhena antidysenterica and Viola canescens, plants traditionally used against malaria in the Garhwal region of north-west Himalaya. Malar J 10:20. https://doi.org/10.1186/1475-2875-10-20

Wachstein M, Meisel E (1957) Histochemistry of hepatic phosphatases of a physiologic pH; with special reference to the demonstration of bile canaliculi. Am J Clin Pathol 27:13–23

Zahara K, Panda SK, Swain SS, Luyten W (2020) Metabolic diversity and therapeutic potential of holarrhena pubescens: An important ethnomedicinal plant. Biomolecules 10:1–28. https://doi.org/10.3390/biom10091341

Zaletel I, Milutinović K, Bajčetić M, Nowakowski RS (2021) Differentiation of Amyloid Plaques Between Alzheimer’s Disease and Non-Alzheimer’s Disease Individuals Based on Gray-Level Co-occurrence Matrix Texture Analysis. Microsc Microanal 27:1146–1153. https://doi.org/10.1017/S1431927621012095

Click here to access/download;Figure;Fig 1.tif

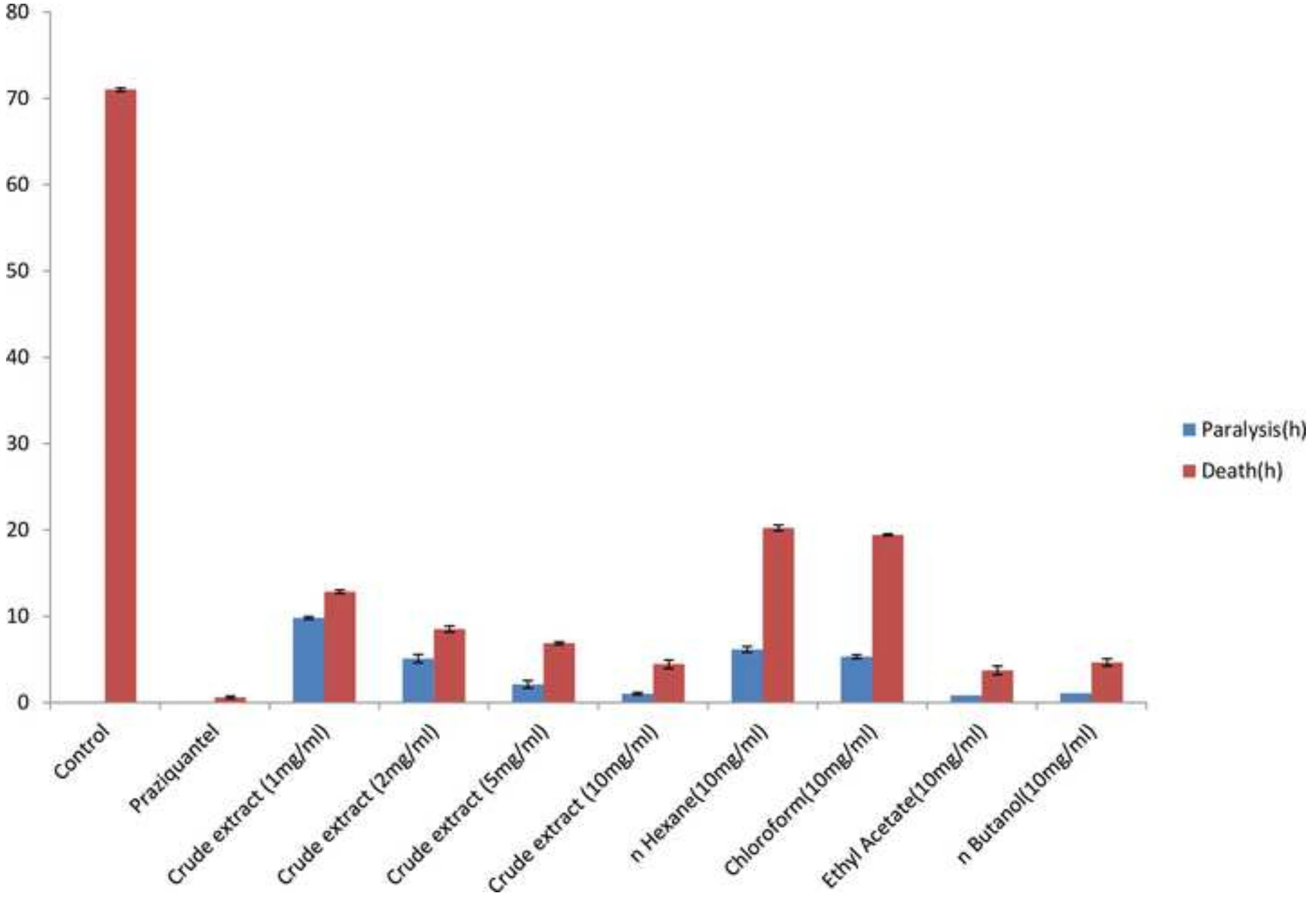

Figure

Click here to access/download;Figure;Fig 3.tif

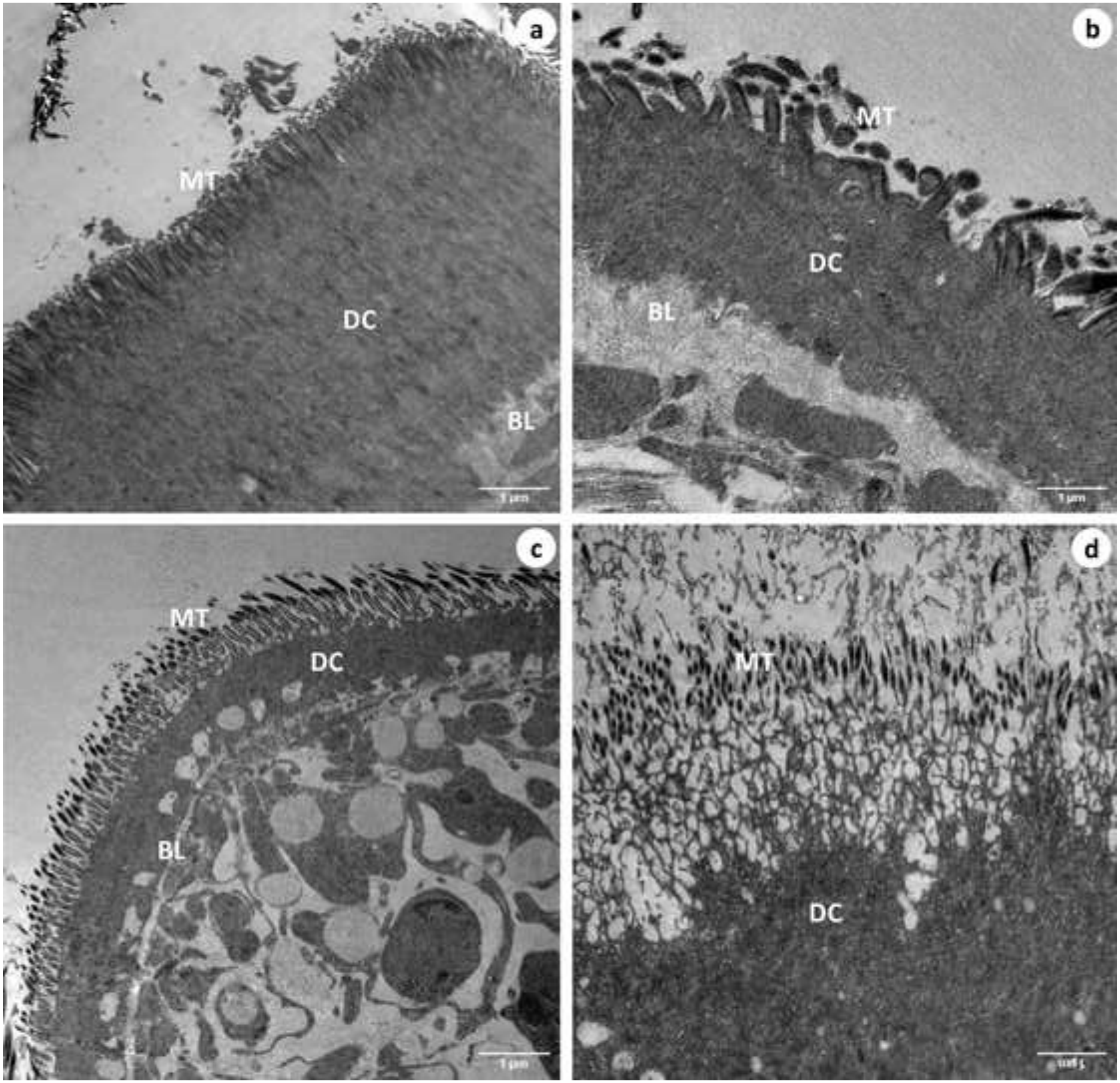

Figure Click here to access/download;Figure;Fig 4.tif

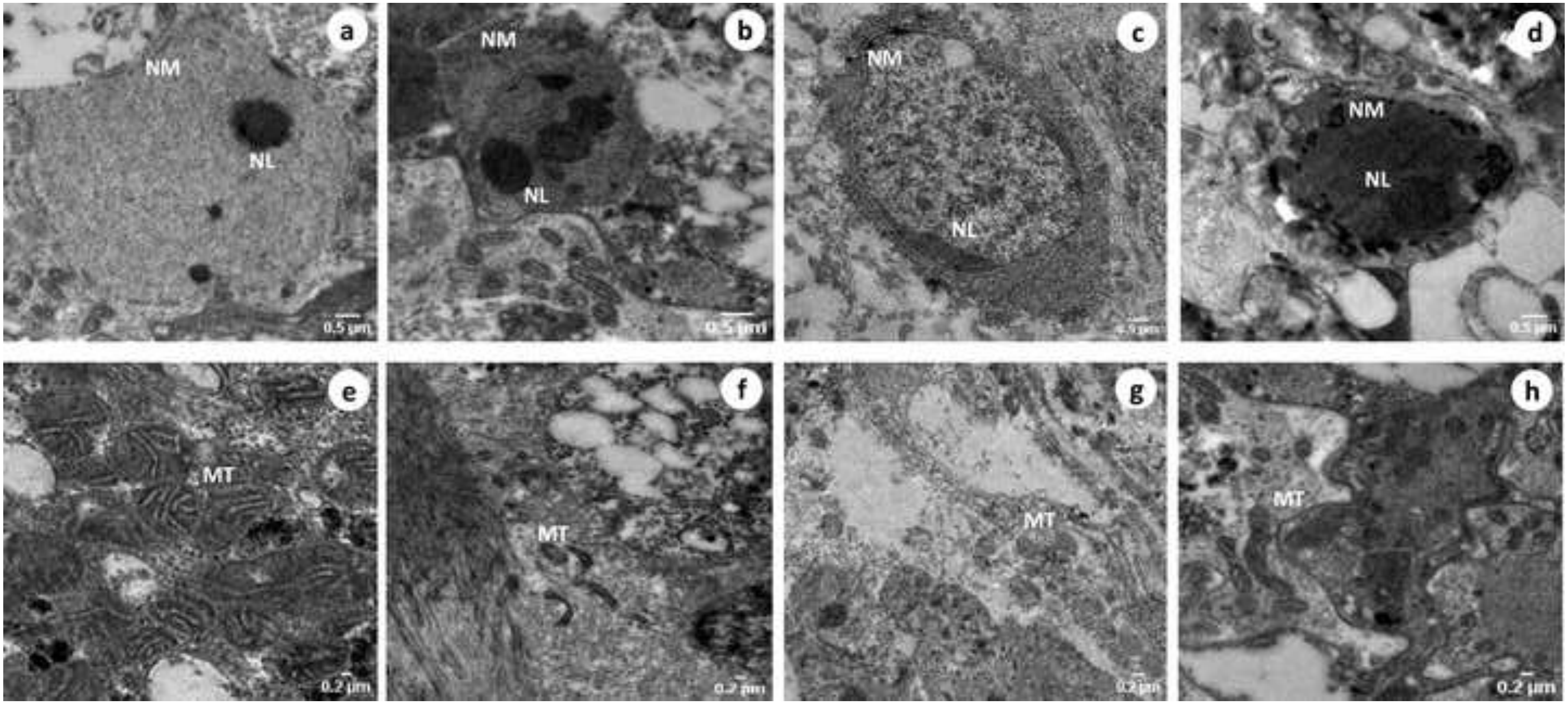

Figure

Click here to access/download;Figure;Fig 2.tif

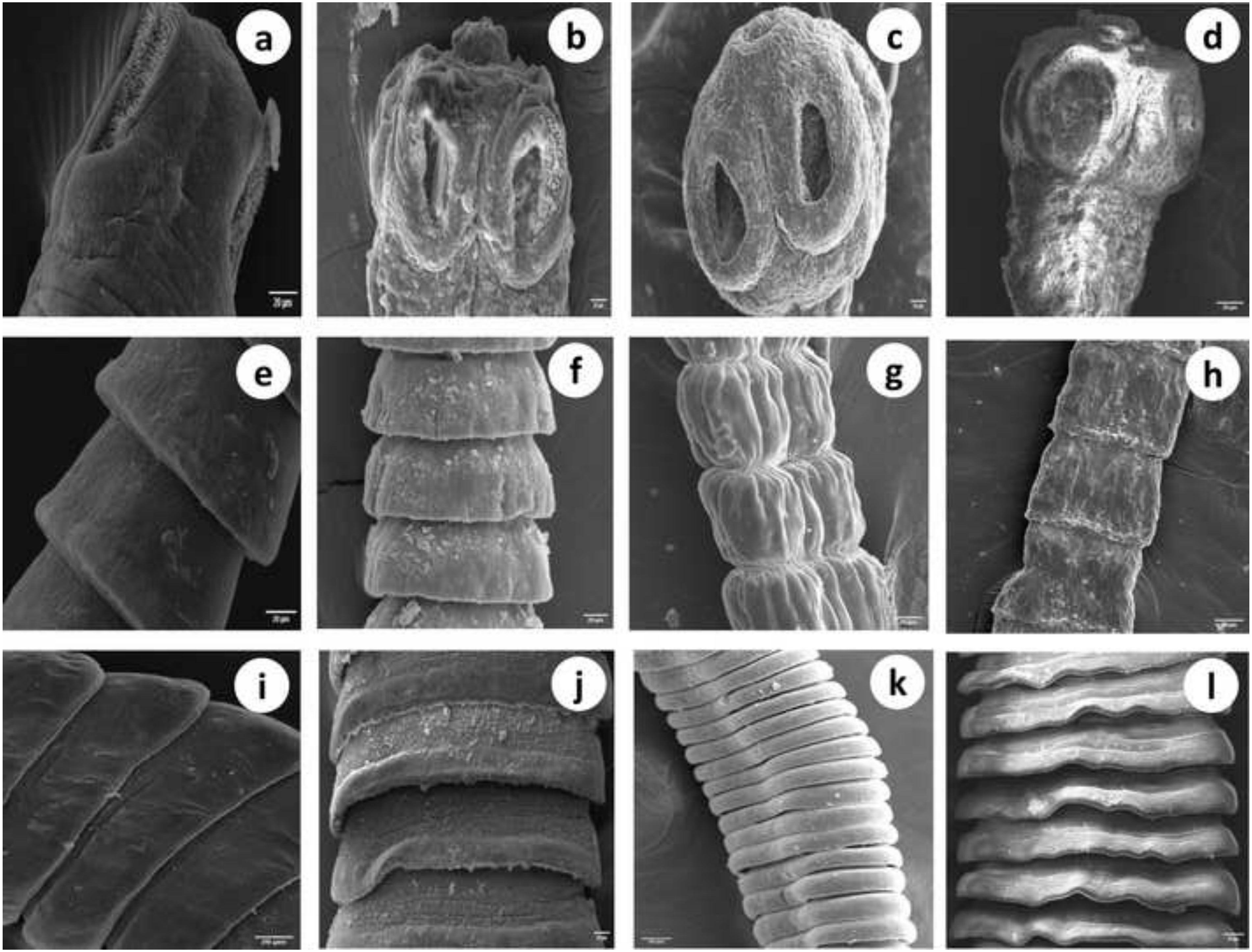

Figure

Click here to access/download;Figure;Fig 6.tif

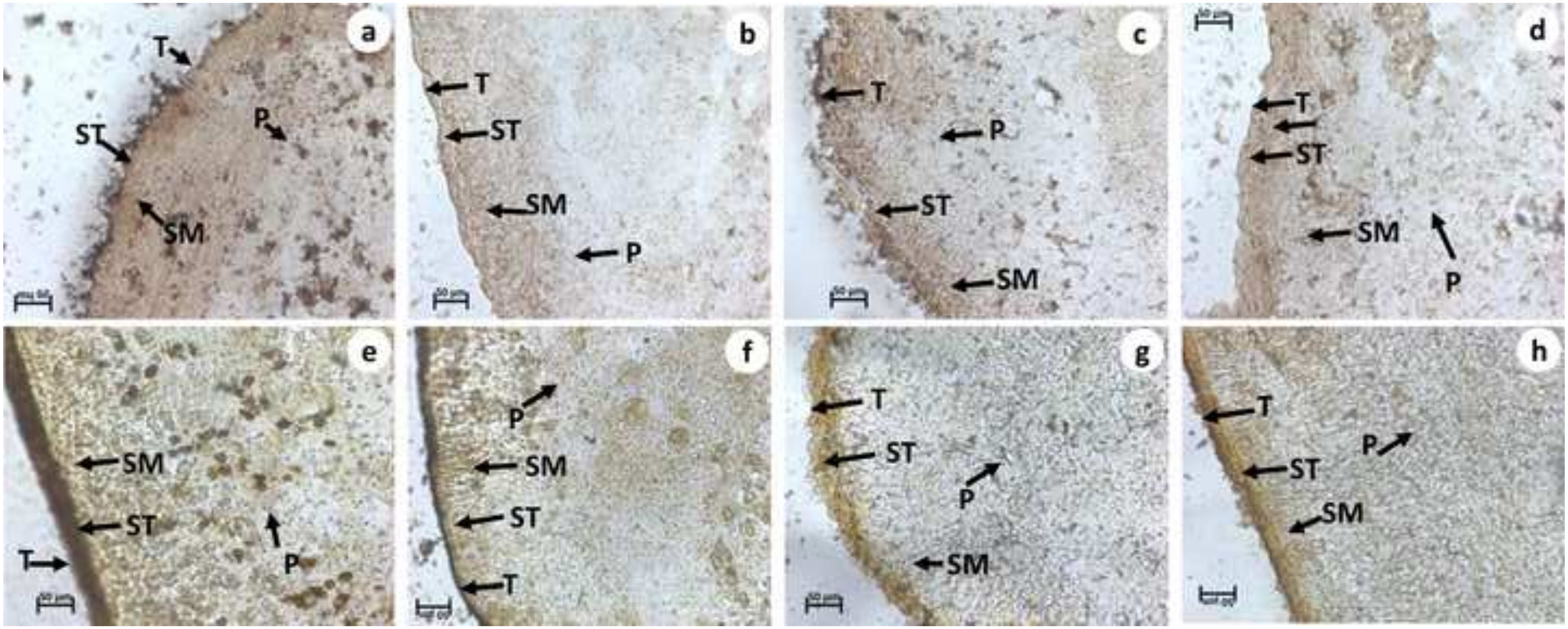

Figure

Click here to access/download;Figure;Fig 5.tif

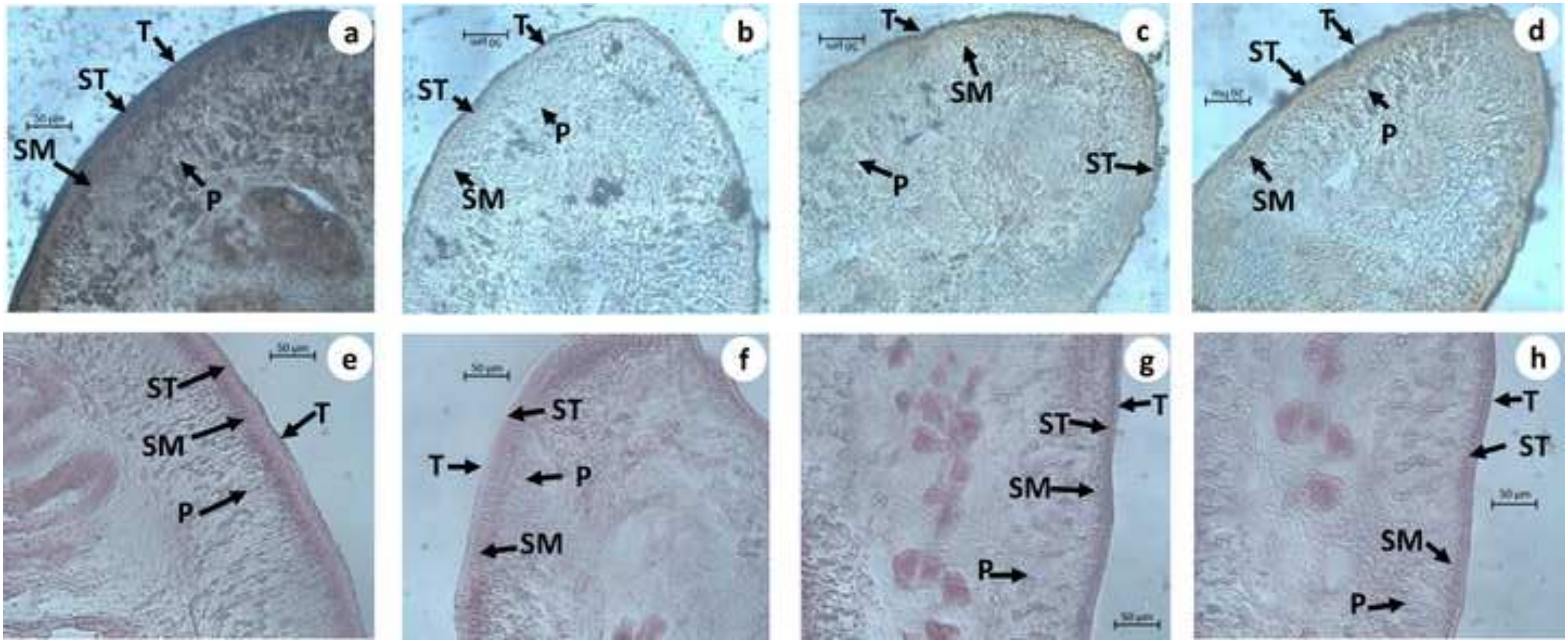

Figure

Click here to access/download;Figure;Fig 8.tif

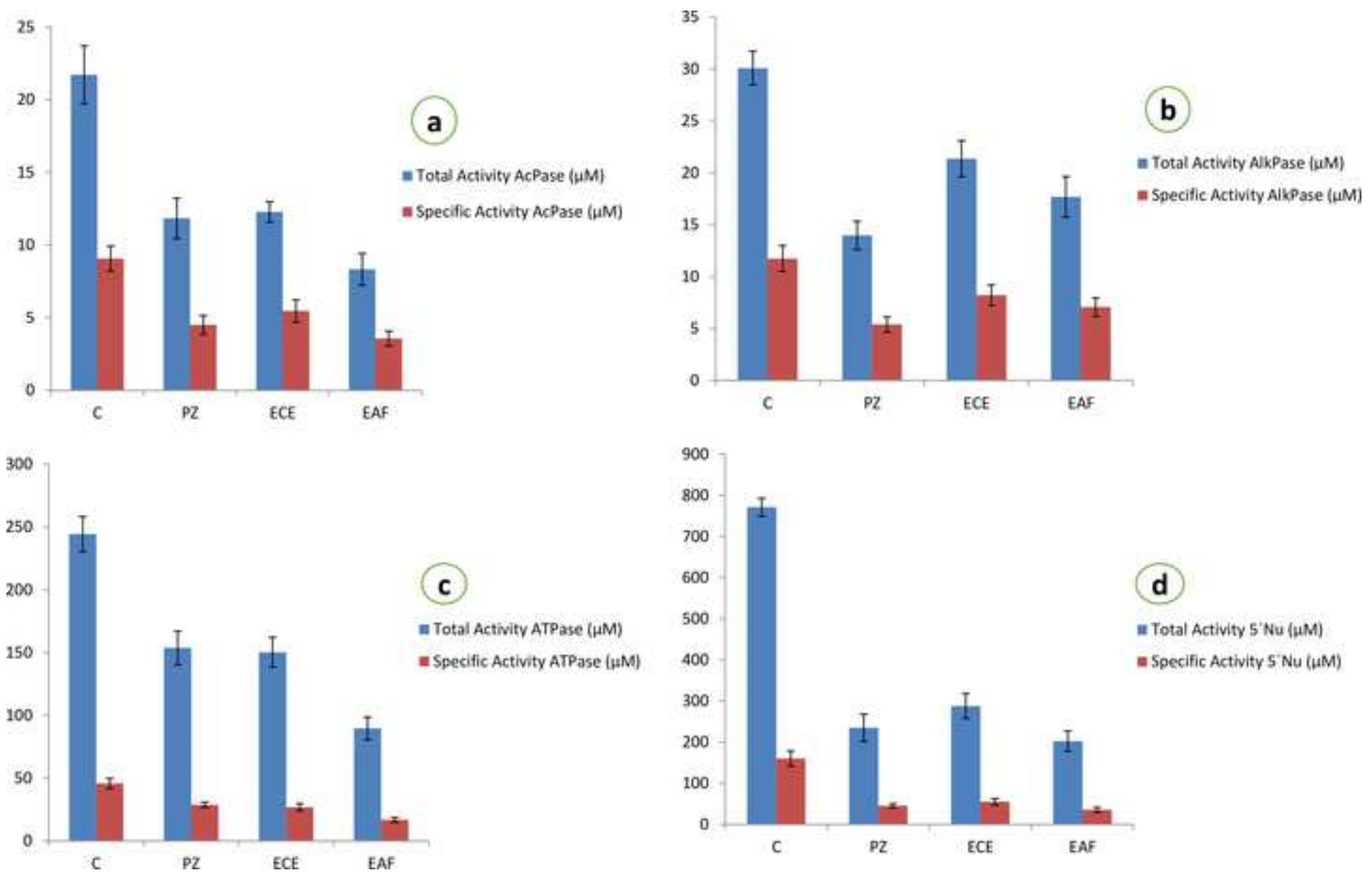

Figure Click here to access/download;Figure;Fig 7.tif

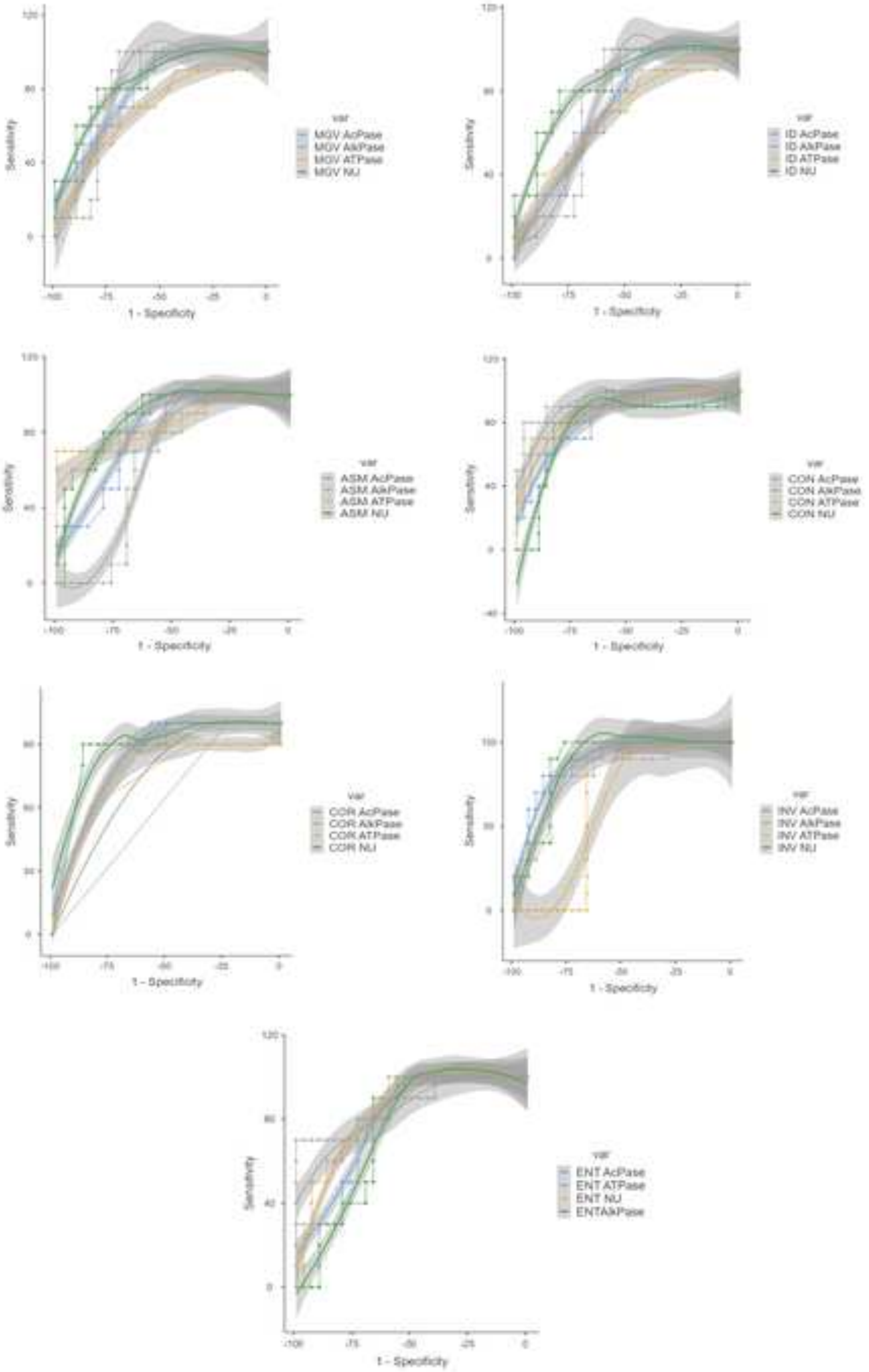

Supplement: Attachment — Submitted filename: PONE-D-23-03097_review-er-revised.pdf [file pone.0282033.s005.pdf]
