# Supplementary material for: Anthelmintic efficacy of Holarrhena pubescens against Raillietina spp. of domestic fowl through ultrastructural, histochemical, biochemical and GLCM analysis
Source: PLoS One. 2023 Sep 14;18(9):e0282033. doi: 10.1371/journal.pone.0282033 (PMC10501554; doi:10.1371/journal.pone.0282033)
Supplement: Attachment — Submitted filename: Rebuttal Letter.pdf [file pone.0282033.s006.pdf]

Dear Sir,

With respect to the comments made by the reviewers for our submitted article "Anthelmintic efficacy of *Holarrhena pubescens* against *Raillietina* spp. of domestic fowl through ultrastructural, histochemical, biochemical and GLCM analysis" I would like to submit our rebuttal as under.

1. Please ensure that your manuscript meets PLOS ONE's style requirements, including those for file naming. The PLOS ONE style templates can be found at

https://journals.plos.org/plosone/s/file?id=wjVg/PLOSOne_formatting_sample_main_body.pdf and https://journals.plos.org/plosone/s/file?id=ba62/PLOSOne_formatting_sample_title_authors_affiliations.pdf

Response:

All the style requirements of PLOS ONE including those for file naming have been met with in accordance to the links provided.

2. Please note that PLOS ONE has specific guidelines on code sharing for submissions in which author-generated code underpins the findings in the manuscript. In these cases, all author-generated code must be made available without restrictions upon publication of the work.

Please review our guidelines at https://journals.plos.org/plosone/s/materials-and-software-sharing#loc-sharing-code and ensure that your code is shared in a way that follows best practice and facilitates reproducibility and reuse.

Response:

DOI 10.1101/2023.02.07.527541

3. Please provide the full species name and source for the worms used.

Response:

Two species of *Raillietina* occur mostly in the study area namely *R.echinobothrida* and *R. tetragona* with the prevalence of *R.echinobothrida* being higher according to our observations. However, since the sample worms obtained were not segregated according to species during the study, we preferred to use *Raillietina* spp. in denoting the model organism.

The worms were obtained after dissecting the intestines of freshly slaughtered local fowl (*Gallus gallus domesticus*) procured from the local market of Cooch Behar, West Bengal, India. We have mentioned the same in the Materials and Methods section, line 148-150.

4. In your Data Availability statement, you have not specified where the minimal data set underlying the results described in your manuscript can be found. PLOS defines a study's minimal data set as the underlying data used to reach the conclusions drawn in the manuscript and any additional data required to replicate the reported study findings in their entirety. All PLOS journals require that the minimal data set be made fully available. For more information about our data policy, please see http://journals.plos.org/plosone/s/data-availability.

Upon re-submitting your revised manuscript, please upload your study's minimal underlying data set as either Supporting Information files or to a stable, public repository and include the relevant URLs, DOIs, or accession numbers within your revised cover letter. For a list of acceptable repositories, please see http://journals.plos.org/plosone/s/data-availability#loc-recommended-repositories. Any potentially identifying patient information must be fully anonymized.

Important: If there are ethical or legal restrictions to sharing your data publicly, please explain these restrictions in detail. Please see our guidelines for more information on what we consider unacceptable restrictions to publicly sharing data: http://journals.plos.org/plosone/s/data-availability#loc-unacceptable-data-access-restrictions. Note that it is not acceptable for the authors to be the sole named individuals responsible for ensuring data access.

We will update your Data Availability statement to reflect the information you provide in your cover letter.

Response:

All the minimal data set underlying the results have been uploaded as Supplementary Information files [S1_Table and S1_Fig, S4_Table and S8_Fig, S4_Table and S8_Fig(2)] in the submission portal. There are no ethical or legal restrictions to sharing our data.

5. Please amend either the abstract on the online submission form (via Edit Submission) or the abstract in the manuscript so that they are identical.

Response:

Done via the online submission form.

Additional Editor Comments:

Reviewers have now commented on your paper. You will see that they are advising that you revise your manuscript. If you are prepared to undertake the work required, I would be pleased to reconsider my decision. Moreover, the writing must be seriously improved. In several occasions phrases seems not have connection with the following phrase, besides lack of scientific accuracy in several parts of manuscript and typographic mistakes.

[Note: HTML markup is below. Please do not edit.]

Reviewers' comments:

Reviewer's Responses to Questions

Comments to the Author

1. Is the manuscript technically sound, and do the data support the conclusions?

The manuscript must describe a technically sound piece of scientific research with data that supports the conclusions. Experiments must have been conducted rigorously, with appropriate controls, replication, and sample sizes. The conclusions must be drawn appropriately based on the data presented.

Reviewer #1: Yes

Reviewer #2: Yes

Reviewer #3: Partly

---

2. Has the statistical analysis been performed appropriately and rigorously?

Reviewer #1: Yes

Reviewer #2: Yes

Reviewer #3: N/A

---

3. Have the authors made all data underlying the findings in their manuscript fully available?

The PLOS Data policy requires authors to make all data underlying the findings described in their manuscript fully available without restriction, with rare exception (please refer to the Data Availability Statement in the

manuscript PDF file). The data should be provided as part of the manuscript or its supporting information, or deposited to a public repository. For example, in addition to summary statistics, the data points behind means, medians and variance measures should be available. If there are restrictions on publicly sharing data—e.g. participant privacy or use of data from a third party—those must be specified.

Reviewer #1: Yes

Reviewer #2: Yes

Reviewer #3: Yes

---

4. Is the manuscript presented in an intelligible fashion and written in standard English?

PLOS ONE does not copyedit accepted manuscripts, so the language in submitted articles must be clear, correct, and unambiguous. Any typographical or grammatical errors should be corrected at revision, so please note any specific errors here.

Reviewer #1: Yes

Reviewer #2: Yes

Reviewer #3: No

---

5. Reviewer Comments to the Author

Please use the space provided to explain your answers to the questions above. You may also include additional comments for the author, including concerns about dual publication, research ethics, or publication ethics. (Please upload your review as an attachment if it exceeds 20,000 characters)

<u>Reviewer #1: Comments to the authors of the article:</u>
1- It is recommended that in the introduction part of the article, a short explanation about the properties of chemical compounds and active ingredients of Holarrhena pubescens plant extract is given.

Response:

We have included a short account of the chemical compounds and active ingredients of *Holarrhena pubescens* plant extracts from literature in the introduction section in two paragraphs from line 104-123 in the revised manuscript.

2- Raillietina worms isolated from the intestines of birds survived for how many hours in the culture media and laboratory conditions, and by what index is the boundary between the paralysis of the worms and the complete death of the worms determined?

Response:

The control worms survived for about 72 hours in the culture media and laboratory conditions. The boundary between paralysis and death of the worms were determined by the absence of movement in the paralysed worms when transferred to warm PBS. We have included the description in the Materials and methods section line no. 157-161 in the revised manuscript.

3- It is better for the authors to use more recent references (after 2015) in the article. For example, it is recommended that from the article [Garedaghiyagoob, Khaki, A., Raza, S.H.A., (...), Abdelgayed, S.S., Kakar, M.U. Epidemiological and pathological studies on the helminthic parasites in native chickens of Tabriz city, Iran. Genetics and Molecular Research. 2017; 16(4),gmr16039824. DOI http://dx.doi.org/10.4238/gmr16039824.] to use in them article references.

Response:

As advised, we have included new and recent references in our manuscript. The number of references have increased from 40 to 68 in the revised manuscript.

4- It is recommended that in the materials and methods section of the article, the exact geographical location (for example, the exact latitude and longitude)

of the sampling location of the Holarrhenapubescens plant, which is in the region of West Bengal, India, and also the sampling year should be mentioned.

Response:

Fresh bark was collected from the plant *H. pubescens* from the town of Cooch Behar (26.3169° N, 89.4457° E), West Bengal, India in month of April- June, Since 2018 till date. We have added it in the Materials and method section line no. 134-135 in the revised manuscript.

Reviewer #2: The manuscript "Anthelmintic efficacy of Holarrhena pubescens against Raillietina spp. in domestic fowl through ultrastructural, histochemical, biochemical and GLCM analysis" it is interesting and presents good execution in the methodology. However, I would like the authors to consider the following comments:

- In section: Introduction

The introduction is poor. I suggest describing biological, morphological characters, zoonotic and veterinary importance of Raillietin spp. once infections are highly pathogenic in domestic chickens (Gallus gallusdomesticus), for example.

In addition, reporting the role of man as an accidental host in Raillietinaspp infections. Human infection is rare, but has been reported from Central and South America, Cuba, Iran, Japan, Southeast Asia, the Philippines, French Polynesia, Australia, and the United States (Hawaii).

Please specify the real objective of this study. Why was Raillietina spp. chosen as a study model?

Response:

The introduction section has been restructured considering the comments of the reviewer and all the mentioned points have been included in the revised manuscript.

-In section: Materials and Methods- Experiments- Efficacy Testing, line 86.

Write more details as parasites were exposed to extracts. Petri dish? Culture plates? medium of culture?

Response:

The parasites were cultured in Phosphate Buffer Saline (PBS), pH 7.4 at 37^{0}C in petri dishes in an incubator. We have added this in line no. 154 in Materials and Methods section in the revised manuscript.

-In section: Results

Fig. 1. The control group cause Mortality of *Raillietina* spp. ???

Response:

No, but the worms survived in PBS for a limited period of time i.e.about 71 hours.

Please insert subtitles on the Y axis of the graph (mortality in hours)

Response:

Done as advised.

-In section: Discussion

The discussion is poor. I suggest discussing the importance of morphological changes in Raillietina spp. presented in SEM and MET, on the influence on the parasite- host relationship.

In addition, emphasize the need to develop new therapeutic alternatives in the veterinary parasitology field, since Raillietina spp. it represents such importance.

Response:

The paragraph in the Discussion section line no. 329- 341 has been rewritten to include the points mentioned by the reviewer. Other sections have been rephrased as well as new information has been added to augment the discussion.

Reviewer #3: The reviewed manuscript describes the obtention and study of anthelmintic properties of ethanolic crude extract and their fractions obtained from different solvents from steam bark of Holarrhena pubescens against Raillietina spp. The manuscript affirms the efficacy of extracts base on comparison with Praziquantel (time of paralysis and time of death), besides observation of effects in parasites by Scanning Electron Microscopy, Transmission Electron Microscopy for instance.
The addition of chromatographic profile, dereplication or any other real information about extract and active fractions would improve the manuscript since it does not afford any information about real composition of evaluated extract and fractions.
The manuscript needs considerably improve in terms of scientific accuracy and correction of several typographic mistakes.
The manuscript in the present format is acceptable for publishing in Plos One since the recommendations below should be taken in consideration or answered.

Major corrections:
1. The authors must carefully and judiciously review the writing of manuscript by complete. There are several points that must be rewrite to text become clearer and present scientific accuracy that is needed.

Response:

Many parts of the manuscript have been rephrased as well as additional points have been included as suggested to improve the quality of English as well as clarity of scientific expression in the revised manuscript.

Minor corrections:
1. It would be possible add consistent information of composition of extracts and active fractions of plant species, besides literature data cited?

Response:

Information about the composition of the extracts have been added in the introduction section line no. 104-123 in the revised manuscript from literature. However, data on the phytochemical characterisation of our plant extract is still not presentable though we plan to publish the same in the near future.

2. Plant species can vary easily the chemical composition depending on period of year. Add the date of obtention of plant species.

Response:

2018, added in Materials and Methods section line no. 134-135 of the revised manuscript.

3. Identification of plant species is important to attest it's the real identity?

Please afford the A the nunber of voucher specimen and Herbarium where it was deposited.

Respose.

Dr. Monoranjan Chowdhury identified the plant and the accession number is- 12638. It was kept at herbarium of Department of Botany, University of North Bengal, Darjeeling, West Bengal. Mention in Materials and Methods section line no. 135-137 of the revised manuscript

4. (Line17, 18): The authors say that manuscript focuses on ethanolic extract and ethyl acetate fraction, however fractions obtained from different solvents were evaluated in the efficacy test. As result, EtOH and AcOEt presented more efficacy and then this extracts were subject to following procedures. Please could you explain why n-BuOH was not selected for following tests, since the values are quite similar with EtOH extract (Table 1)?

Response:

The ethanolic extract was first used for efficacy testing. Then the same extract was then subjected to fractionation using hexane, chloroform, ethyl acetate and n-butanol. These four fractions obtained from the ethanolic extract were again used for efficacy testing, out of which the ethyl acetate fraction showed the highest efficacy.

5. (Line 70) Could you explain the observation "(without chlorophyll)"? What does this mean? Any method to remove chlorophyll was used or just to mention that this part of plant is free of chlorophyll?

Response:

No method of removing chlorophyll was used. However, the outer layer of the obtained bark was peeled to obtain the fresh brownish inner layer. Nevertheless, we have removed the phrase "without chlorophyll" from the revised manuscript.

6. (line 72) "The dried bark was processed…". What exactly is processed? Please be clear. This phase as others presents in the manuscript lack of scientific accuracy. Probably, processed means "submitted to extraction". However, the extraction method is described at lines 75, 76, 77 and 78. The authors must rewrite this section aiming a better description of complete process (since obtention of plant material to fraction obtention).

Response:

We have rephrased the mentioned section in the Materials and Methods line no. 135-145 in the revised manuscript to improve the scientific accuracy.

7. (line72) The extraction process took 15-20 days? Could you explain why and describes in detail the complete methodology?

Response:
The dried bark was kept immersed in ethanol for 15-20 days with repeated stirring to maximize the yield of phytochemicals**.**

8. (line 248) In discussion section authors bring to manuscript information about phytochemistry aspects of studied plant species base on literature. However, all these points raised by authors necessarily don't reflect any considerably discussion to paper in this section. These points must be in introduction to present bioactive compounds of studied species. If authors had performed LC-MS/MS analysis of ETOH extract and/or active fractions and dereplication of compounds, a discussion could explored in this section.

Response:

As advised, we have moved the section dealing with the phytochemistry aspects of the plant to the Introduction section line no. 104-123 in the revised manuscript.

We have not performed any LC-MS/MS analysis of the extract yet.

9. (line 306) The section conclusion should be rewrite, since points mentioned don't bring the idea of conclusion of presented work. The ideas should be expanded. I recommend the authors don't use sentences as "The result of various experiments has shown".

Response:

The conclusion has been rewritten as advised.

---

6. PLOS authors have the option to publish the peer review history of their article (what does this mean?). If published, this will include your full peer review and any attached files.

If you choose "no", your identity will remain anonymous but your review may still be made public.

We do not wish to make the peer review history public.

Hope this will suffice for now. We are open for discussion for any further comments made by the reviewers.

Thanking you,

Yours sincerely,

Dr. Pradip Kumar Kar
