# Supplementary material for: Anthelmintic efficacy of Holarrhena pubescens against Raillietina spp. of domestic fowl through ultrastructural, histochemical, biochemical and GLCM analysis
Source: PLoS One. 2023 Sep 14;18(9):e0282033. doi: 10.1371/journal.pone.0282033 (PMC10501554; doi:10.1371/journal.pone.0282033)
Supplement: Attachment — Submitted filename: Rebuttal Letter.pdf [file pone.0282033.s007.pdf]

Dear Sir,

With respect to the comments made by the reviewers for our submitted article "Anthelmintic efficacy of *Holarrhena pubescens* against *Raillietina* spp. Of domestic fowl through ultrastructural, histochemical, biochemical and GLCM analysis" I would like to submit our rebuttal as under.

Additional Editor Comments (if provided):

I recommend that the authors thoroughly revise the text to enhance its English quality. Utilizing the services of a native English speaker would be beneficial in this regard.
Response

Response:

The authors revised the manuscript as and where necessary, and, would like to state that the quality of English of this communication conforms to existing standard comparable to articles published in reputed high-impact journals. The author would like to further reiterate that this manuscript has been written as per convention practiced by Parasitologists, world over, for publication of anthelmintic efficacy data. The corresponding author has already published two articles in Plos One which has been appreciated by the scientific community.

1) **Pradip Kumar Kar**, Sanatan Murmu, Saswati Saha, Veena Tandon, Krishnendu Acharya. Anthelmintic efficacy of gold nanoparticles derived from phytopathogenic fungi *Nigrospora oryzae*. **Plos One** (2014) 9(1): e84693, ISSN No. – 1932-6203, January 2014

2) Anirban Ash, Tomáš Scholz, Alain de Chambrier, Jan Brabec, Mikuláš Oros, **Pradip Kumar Kar**, Shivaji Prabhakar Chavan, Jean Mariaux. Revision of *Gangesia* (Cestoda: Proteocephalidea) in the Indomalayan Region: morphology, molecules and surface ultrastructure. **Plos One** (2012) 7(10): 1-28, e46421 ISSN No. – 1932-6203, October 2012

Hence, the authors would be highly obliged if this explanation suffices the quandary on the quality of English of this article, and would like to provide further clarification / justification if so desired by the editorial team.
[Note: HTML markup is below. Please do not edit.]

Reviewers' comments:

Reviewer's Responses to Questions

Comments to the Author

1. If the authors have adequately addressed your comments raised in a previous round of review and you feel that this manuscript is now acceptable for publication, you may indicate that here to bypass the "Comments to the Author" section, enter your conflict of interest statement in the "Confidential to Editor" section, and submit your "Accept" recommendation.

Reviewer #1: All comments have been addressed

Reviewer #3: (No Response)

---

2. Is the manuscript technically sound, and do the data support the conclusions?

The manuscript must describe a technically sound piece of scientific research with data that supports the conclusions. Experiments must have been conducted rigorously, with appropriate controls, replication, and sample sizes. The conclusions must be drawn appropriately based on the data presented.

Reviewer #1: Yes

Reviewer #3: Yes

---

3. Has the statistical analysis been performed appropriately and rigorously?

Reviewer #1: I Don't Know

Reviewer #3: Yes

---

4. Have the authors made all data underlying the findings in their manuscript fully available?

The PLOS Data policy requires authors to make all data underlying the findings described in their manuscript fully available without restriction, with rare exception (please refer to the Data Availability Statement in the manuscript PDF file). The data should be provided as part of the manuscript or its supporting information, or deposited to a public repository. For example, in addition to summary statistics, the data points behind means, medians and variance measures should be available. If there are restrictions on publicly sharing data—e.g. participant privacy or use of data from a third party—those must be specified.

Reviewer #1: Yes

Reviewer #3: Yes

---

5. Is the manuscript presented in an intelligible fashion and written in standard English?

PLOS ONE does not copyedit accepted manuscripts, so the language in submitted articles must be clear, correct, and unambiguous. Any

typographical or grammatical errors should be corrected at revision, so please note any specific errors here.

Reviewer #1: Yes

Reviewer #3: Yes

---

6. Review Comments to the Author

Please use the space provided to explain your answers to the questions above. You may also include additional comments for the author, including concerns about dual publication, research ethics, or publication ethics. (Please upload your review as an attachment if it exceeds 20,000 characters)

Reviewer #1: it is recommended that from the article [Garedaghiyagoob, Khaki, A., Raza, S.H.A., (...), Abdelgayed, S.S., Kakar, M.U. Epidemiological and pathological studies on the helminthic parasites in native chickens of Tabriz city, Iran. Genetics and Molecular Research. 2017; 16(4),gmr16039824. DOI http://dx.doi.org/10.4238/gmr16039824.] to use in them article references.

Response:

It has been done. Line number: 72

Reviewer #3: The authors improved the manuscript compared to the first version. However, it is necessary several adjustments in the text taking into consideration typographic mistakes and phrases that must be rewritten to let the text concise and fluid which is necessary for a scientific paper. Unfortunately, the section that describes the extraction procedure, as well as the obtention of organic phases, remains without details. Even though the focus of the work is the evaluation of anthelmintic properties of extract and organic phases of H. pubescens, details of the obtention of these materials are required and essential. As a final consideration, it would be helpful to submit the text to a scientific-technical translation company to adjust the manuscript to be published.
Bellow follows specific comments and considerations to the authors.

Minor corrections:
1. Please, maintain a solvent nomenclature standardized. Use the first letter capitalized for all of them or none of them (except at the beginning of a phrase, for instance);

Response:

Done as advised.

2. Use italics for “n” before “-butanol”.

Response:

Done as advised.

3. Please, pay attention to spaces between characters, punctuation, and hyphen. Several points must be reviewed.

Response:

Done as advised.

4. The same aspect is seen for units after numbers. Please follow a pattern and check spaces between numbers and units.

Response:

Done as advised.

5. Lines 143 and 144 – Unfortunately the procedure described is not clear enough. Please be specific as much as you can.

Response:

50 ml of aqueous crude extract solution (2.5 grm in 50 ml distilled water) was combined with 50 ml of Hexane and left to stand for 2 hours. The Hexane fraction was subsequently removed from the upper phase using the liquid liquid separation technique. The method was repeated three times using Chloroform (lower phase), Ethyl acetate, and n-Butanol. The final volume of each fraction (150 ml) was collected and dried using a Rotary Vacuum Evaporator (Buchi Rotavapor R-100). Around 0.25 g, 0.14 g, 0.92 g and 1.1 g of Hexane, Chloroform, Ethyl acetate and *n*-Butanol fractions were obtained from 2.5 g of crude extract respectively. Mentioned in Materials and Methods section line no. 143-148 of the revised manuscript

6. Lines 143 and 144 – It is necessary to inform the right procedure of liquid-liquid extraction as requested previously. Be specific in terms of the used mass of crude extract, how this crude extract was solubilized (what solvent), or partially solubilized, the addition order of solvents, quantities of used solvents the final mass of each phase obtained, for instance.

Response:

Mentioned in Materials and Methods section line no. 143-148 of the revised manuscript

7. Is not completely clear how and which procedures (liquid-liquid or fractional distillation) were employed or in which stage each of them was used.

Response:

Mentioned in Materials and Methods section line no. 143-148 of the revised manuscript

8. Line 144 – Could you explain how fractional distillation was employed to obtain the organic phases?.

Response:

Mentioned in Materials and Methods section line no. 143-148 of the revised manuscript

9. Despite information about the chemistry of plant species was included in the introduction, the text must be reviewed from lines 100 to 122 to become more concise and let the text fluid. The whole idea of this part is impaired by the repetition of ideas and phrases that should appear in a better position to bring consistency to the text.

Response:

Done as advised.

10. Line 111 – Please, replace "Kutaja" with the scientific name of the plant species.

Response:

Done as advised.

11. Lines 114 to 117 – Please, include the reference about alkaloids in H. pubescens.

Response:

Included in line number 115.

12. From lines 134 to 137 – This part of the text needs to be rewritten to bring fluidity.

Response:

Done as advised.

13. In many parts of the manuscript, the authors don't let clear the amount of material or time of procedure used. For example, lines 137, 138, 141, and other parts. Why is not shown the exact amount of material and time used?.

Response:

The plant materials were finely chopped and air dried for 24-36 hours after being collected. The dried bark components were soaked in ethanol in a glass container (100 grams in 500 ml) for 15-20 days with frequent stirring. The solution was then filtered using Whatman Filter Paper (No. 14), and then dried using a Rotary Vacuum Evaporator (Buchi Rotavapor R-100). After drying, the collected crude extracts of plant material were stored under refrigeration at 4°C until further use. Around 7.0-8.0 gram (g) of plant extract was obtained from 100 g of plant material soaked in 500 millilitre (ml) ethanol.

It has been clearly mentioned in the main manuscript earlier, line number 135 -142 that only stem bark is used for crude extract preparation and other subsequent experiments.

14. Line 141 – As a suggestion replace "stored in a container at" with "stored under refrigeration at".

Response:

Done as advised.

15. Line 155 mentions a 20 mg/mL concentration in the row of samples tested for efficacy testing. However, it is not seen this concentration in Table 1. Please add the value in the table or remove the information (line 155).

Response:

Removed the information.

---

7. PLOS authors have the option to publish the peer review history of their article (what does this mean?). If published, this will include your full peer review and any attached files.

If you choose "no", your identity will remain anonymous but your review may still be made public.

Response:

We do not wish to make the peer review history public.

Hope this will suffice for now. We are open for discussion for any further comments made by the reviewers.

Thanking you,

Yours sincerely,

Dr. Pradip Kumar Kar
